# Correlation analysis of hemoglobin, albumin, lymphocyte, platelet score and platelet to albumin ratio and prognosis in patients with lung adenosquamous carcinoma

**DOI:** 10.3389/fonc.2023.1166802

**Published:** 2023-09-07

**Authors:** Tiantian Zhang, Wei Liu, Chunhua Xu

**Affiliations:** ^1^ Department of Respiratory Medicine, Affiliated Nanjing Brain Hospital, Nanjing Medical University, Nanjing, Jiangsu, China; ^2^ Clinical Center of Nanjing Respiratory Diseases and Imaging, Nanjing Chest Hospital, Nanjing, Jiangsu, China

**Keywords:** lung adenosquamous carcinoma, surgery, PAR, HALP score, differentiation degree, prognosis

## Abstract

**Objective:**

To investigate the effect of hemoglobin, albumin, lymphocytes, platelet (HALP) score and platelet to albumin ratio (PAR) on prognosis of patients with lung adenosquamous carcinoma (ASC) after surgery.

**Patients and methods:**

A total of 52 patients diagnosed with ASC after surgical resection were collected from Nanjing Chest Hospital from 2012 to 2021, and their general clinical data, pathological data and laboratory indexes were collected. The changes of Alb and Plt levels before and after surgery, HALP scores (hemoglobin albumin lymphocytes/platelets), and postoperative PAR, PLR, NLR were retrospectively analyzed, and their influence on the prognosis of patients with ASC was investigated. The cut-off value of △Alb, △Plt, postoperative PAR, PLR and NLR were determined by the receiver operating characteristic (ROC) curve, the optimal cut-off value of HALP score before and after surgery was calculated by using X-tile software, and the clinicopathological characteristics were compared between the high PAR and low PAR groups and between high HALP score and low HALP score group to analyze the factors influencing the prognosis of patients with ASC. Univariate and multivariate Cox proportional regression analyses were used to assess independent risk factors affecting overall survival (OS) and disease-free survival (DFS) in patients with ASC. Kaplan-Meier method was used to evaluate the correlation between OS, DFS and PAR and HALP score.

**Results:**

A critical value of PAR was 7.40×10^9 and an area under the curve (AUC) of 0.737 (95%CI: 0.597-0.876, *P* = 0.004). The best cut-off value of the preoperative HALP score was 24.3. Univariate Cox analysis showed that the cut margin (*P* = 0.013), the degree of differentiation (*P* = 0.021), N stage (*P* = 0.049), △Plt (*P* = 0.010), △Alb (*P* = 0.016), PAR (*P* = 0.003), NLR (*P* = 0.025), PLR (*P* = 0.029), preoperative HALP score (*P* = 0.000) and post-operative HALP score (*P* = 0.010) were all associated with postoperative OS in ASC patients. Cut margin (*P* = 0.029), the degree of differentiation (*P* = 0.045), maximum tumor diameter (*P* = 0.018), N stage (*P* = 0.035), △Plt (*P* = 0.007), △Alb (*P* = 0.007), PAR (*P* = 0.004), NLR (*P* = 0.041), PLR (*P* = 0.030), preoperative HALP score (*P* = 0.000), and postoperative HALP score (*P* = 0.011) were related to postoperative DFS in ASC patients. Multivariate analysis revealed that PAR (HR: 6.877, 95%CI: 1.817-26.038, *P* = 0.005), differentiation degree (HR: 0.059, 95%CI: 0.006-0.591, *P* = 0.016) and preoperative HALP score (HR: 0.224, 95%CI: 0.068-0.733, *P* = 0.013) had significant effect on OS. Tumor maximum diameter (HR: 3.442, 95%CI: 1.148-10.318, *P* = 0.027) and preoperative HALP score (HR: 0.268, 95%CI: 0.085-0.847, *P* = 0.025) had significant influence on DFS.

**Conclusion:**

PAR and preoperative HALP score were potentially useful biomarkers for evaluating the outcome of patients with postoperative ASC. PAR, the degree of differentiation and preoperative HALP score were independent prognostic factors for postoperative OS in ASC patients. Maximum tumor diameter and preoperative HALP score were independent prognostic factors for postoperative DFS in ASC patients.

## 1 Introduction

Lung cancer is the most common primary lung malignancy and the leading cause of cancer-related death worldwide (1). According to histopathological classification, lung cancer can be divided into two categories: non-small cell lung cancer (NSCLC) and small cell lung cancer. NSCLC is the most common, accounting for about 85% of the total incidence of lung cancer, including adenosquamous carcinoma of the lung (ASC), lung adenocarcinoma (AC) and squamous cell carcinoma (SCC) (2). ASC is composed of histology components of SCC and AC, each component accounts for at least 10% of the tumor (3). It is a rare histological subtype of NSCLC, accounting for only 0.4%-4% of lung cancer (4). It is characterized by high malignancy, easy early metastasis, rapid progression and poor prognosis, etc. A study by Maeda et al. represented that patients with ASC had worse prognosis than patients with simple AC and SCC, with 5-year survival rates of 23.3%, 58.0% and 40.8%, respectively (5). ASC is not a simple mixture of SCC and AC, and its preoperative diagnosis is difficult. Pathological specimens of surgical excision is the most effective means to diagnose ASC. In recent years, with the improvement of diagnostic level, the incidence of ASC has gradually increased. Clinicopathologic variables such as pathological subtype, gender and TNM stage have been found to be prognostic biomarkers of ASC after surgical resection (6).

In recent decades, systemic inflammatory states have become an important marker of malignant tumors and are closely related to the initiation, progression, metastasis and drug resistance of drug therapy (7). Platelets, as an important link in inflammation, play a crucial role in cancer progression and inflammation. Tumor cells can activate migration of immune cells to tumor sites, thus promoting tumor growth, blood vessel formation and metastasis (8). In addition, as an indicator of the nutritional state of the body, albumin has a variety of physiological functions, including maintaining plasma osmotic pressure, tissue growth and repair, transporting endogenous and exogenous compounds such as various drugs or nutrients, and regulating systemic inflammation. Hypoalbuminemia is easy to lead to postoperative complications, promote tumor growth and migration, and lead to infection and inflammation of patients, thus further aggravating the prognosis of patients (9). PAR, as a derived inflammatory indicator, is a new prognostic immune biomarker. A recent study by Guo et al. showed that a high level of PAR was associated with clinicopathological features and prognosis of NSCLC (10). In addition, Shi et al. found that PAR was an independent risk factor for postoperative OS in patients with gastric cancer (11). Hemoglobin is the main molecule in the body that carries oxygen and transports oxygen. Hypoxia is an important factor in tumor metabolism, survival, invasion, migration, angiogenesis, and resistance to chemotherapy or immunotherapies (12). Tomita et al. retrospectively analyzed the effect of preoperative hemoglobin level on their survival of 240 NSCLC patients, and the 5-year survival rate of normal and low hemoglobin levels were 99.73% and 47.2%, respectively (*P* < 0.0001), indicating that preoperative hemoglobin level was a prognostic factor in NSCLC patients (13). Lymphocytes are the main functional cells of the body’s immune response. In the development of tumors, as a heterogeneous antigen, they can stimulate the body to produce an immune response and produce a large number of lymphocytes. When tumors undergo immune escape, tumor cells can express the antigens that inhibit immune cells, which leads to apoptosis of immune cells after binding with them (14, 15). The HALP score, calculated based on hemoglobin, albumin, lymphocyte and platelet counts, reflecting the patient’s nutritional status and immune status, was first introduced by Chen et al. in 2015 (16), whose study showed that HALP score was closely related to clinicopathological features and was an independent prognostic factor for gastric cancer. In recent years, it has been shown that HALP score is also associated with the prognosis of metastatic RCC. Ekinci et al. found that the mean OS of patients with metastatic renal cell carcinoma with a low HALP score was 17.7 months and the OS was 89.7 months in patients with high HALP score (*P* = 0.001), indicating that OS was shorter in patients with lower HALP score in metastatic renal cell carcinoma (17). However, whether PAR and HALP score may serve as prognostic factors for ASC remains unclear and this study aimed to investigate the clinical significance of inflammatory markers in patients with ASC and their relationship with OS and DFS in ASC patients.

## 2 Materials and methods

### 2.1 Patients

This study included 52 patients with NSCLC who underwent surgical resection in Nanjing Chest Hospital between 2012 and 2021. Inclusion criteria: (a) further diagnosis of ASC by pathology and immunohistochemistry; (b) no other malignant neoplasms; (c) preoperative blood test indexes and postoperative follow-up data were complete. Exclusion criteria: (a) accompanied by preoperative conditions affecting albumin or platelet expression, such as infection, inflammation, hematological diseases, autoimmune diseases, liver or kidney dysfunction; (b) patients with incomplete clinical information or lost to follow-up. This study was approved by the Ethics Committee of Nanjing Brain Hospital and was carried out in accordance with the national law and the current revised Declaration of Helsinki. Informed consent was obtained from all participants in the study.

### 2.2 Data collection

General clinical data (including gender, age, symptoms at medical visit, smoking history, and preoperative chest CT) and pathological data (including the tumor location, tumor maximum diameter, degree of differentiation, margin, and pTNM stage) of patients were collected through the pre-electronic system. Blood routine, blood coagulation routine and blood biochemistry, including platelet count, neutrophil count, lymphocyte count, albumin count, and hemoglobin count were collected before and after surgery. The albumin difference (△Alb) before and after surgery, platelet difference (△Plt) before and after surgery, and platelet to albumin ratio (PAR), neutrophil to lymphocyte ratio (NLR), platelet to lymphocyte ratio (PLR) and hemoglobin (g/L) × albumin (g/L) × lymphocytes (/L)/platelets (/L) (HALP score) were calculated.

### 2.3 Follow-up

Follow-up was conducted by referring to patient medical records and contacting patients by telephone through the pre-electronic system. The endpoint was set as OS, disease-free survival (DFS), or the end date of follow-up (August 05, 2022). OS was defined as the time from the date of the first operation to the date of patient death or the end date of follow-up, and DFS was defined as the time from the date of first surgery to the patient recurrence or the patient died due to disease progression.

### 2.4 Statistical analysis

Statistical analysis of the data was performed using SPSS software version 25.0 and X-tile software version 3.6.1. The critical value of △Alb, △Plt, PAR, NLR and PLR were calculated by drawing ROC curves. The optimal cut-off of the HALP score before and after surgery was calculated using the X-tile. OS and DFS used the Kaplan-Meier method with parallel Log-rank test, and Cox proportional risk model was used for univariate and multivariate analysis. *P* < 0.05 was considered to be a statistically significant difference.

## 3 Results

### 3.1 Clinicopathologic characteristics

A total of 64 patients with ASC undergoing surgical treatment were initially included in this study. Of these, 12 were excluded (5 with thymic squamous cell carcinoma, 7 with missing follow-up or incomplete data), and 52 ASC patients were included in the final analysis. The study included 17 women (32.7%) and 35 men (67.3%), with an age from 43 to 79 years. 25 patients (48.1%) had a history of smoking. In 34 cases (65.4%) the lesion was located in the upper lobe of the lung. In forty-seven cases (90.4%) were margin-negative. 31 patients (59.6%) were stage IA1-IIA, and 21 patients (40.4%) were stage IIB-IIIB. Histological differentiation was poor in 41 patients (78.8%), and lymph node metastasis occurred in 16 patients (30.8%). The OS rate was 53.8%, the DFS rate was 42.3%. Detailed general clinical characteristics are shown in **Table 1**.

**Table 1:** Baseline characters of the 52 ASC patients.

Variables	N (%)
Gender	
Male	35 (67.3)
Female	17 (32.7)
Age at diagnosis (years)	
≤ 60	18 (34.6)
> 60	34 (65.4)
Smoking history	
Yes	25 (48.1)
No	27 (51.9)
Maximum tumor diameter (cm)	
≤ 4	34 (65.4)
> 4	18 (34.6)
Margin	
negative	47 (90.4)
positive	5 (9.6)
T stage	
T1a-T2a	29 (55.8)
T2b-T4	23 (44.2)
N stage	
N0	36 (69.2)
N1	13 (25.0)
N2+N3	3 (5.8)
pTNM stage	
IA1-IIA	31 (59.6)
IIB-IIIB	21 (40.4)
Differentiation	
Poor	41 (78.8)
Moderate-poor	11 (21.2)
Tumor site	
Upper	34 (65.4)
Lower middle	18 (34.6)
Survival	
Yes	28 (53.8)
No	24 (46.2)
Recurrent or Metastatic	
Yes	30 (57.7)
No	22 (42.3)
△Alb	
≤ -5.1g/L	32 (61.5)
> -5.1g/L	20 (38.5)
△Plt	
≤ 62×10^9/L	30 (57.7)
> 62×10^9/L	22 (42.3)
PAR	
≤ 7.40×10^9	26 (50.0)
> 7.40×10^9	26 (50.0)
PLR	
≤ 176.54	16 (30.8)
> 176.54	36 (69.2)
NLR	
≤ 3.61	14 (26.9)
> 3.61	38 (73.1)
Preoperative HALP score	
≤ 24.3	6 (11.5)
> 24.3	46 (88.5)
Postoperative HALP score	
≤ 19.8	28 (53.8)
> 19.8	24 (46.2)

ASC, lung adenosquamous carcinoma; pTNM, pathological tumor, node, metastasis staging; △Alb, the albumin difference before and after surgery; △Plt, the platelet difference before and after surgery; PAR, platelet to albumin ratio; PLR, platelet to lymphocyte ratio; NLR, neutrophil to lymphocyte ratio; preoperative HALP score, preoperative hemoglobin, albumin, lymphocyte, and platelet score; postoperative HALP score, postoperative hemoglobin, albumin, lymphocyte, and platelet score.

### 3.2 Optimal cut-off value for ROC curve

**Figure 1:**
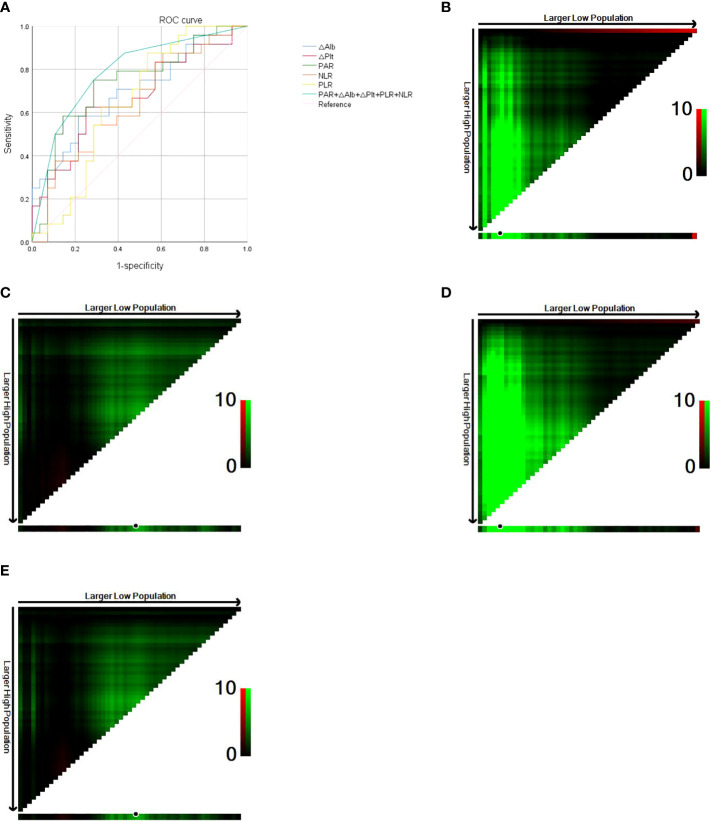
**(A)** Roc curve of PAR, △Plt, △Alb, PLR, NLR. **(B)** Optimal cut-off value for preoperative HALP score (OS of 42 months as the cut-off). **(C)** Optimal cut-off value for postoperative HALP score (OS of 42 months as the cut-off). **(D)** Optimal cut-off value for preoperative HALP score (DFS of 36 months as the cut-off). **(E)** Optimal cut-off value for postoperative HALP score (DFS of 36 months as the cut-off).

According to the ROC curve analysis, the cut-off value of postoperative PAR in ASC patients was 7.40 × 10^9, and the area under the curve (AUC) was 0.737 (sensitivity 75.0%, specificity 71.4%, *P* = 0.004, **Figure 1A**). The cut-off value of △Plt was 62 × 10^9, and the AUC was 0.670 (sensitivity 62.5%, specificity 75.0%, *P* = 0.036, **Figure 1A**). The cut-off value of △Alb was -5.1, and the AUC was 0.700 (sensitivity 58.3%, specificity 78.6%, *P* = 0.014, **Figure 1A**). The postoperative PLR cut-off value was 176.54 and the AUC was 0.650 (sensitivity 87.5%, specificity 46.4%, *P* = 0.064, **Figure 1A**). The postoperative NLR cut-off was 3.61 and the AUC was 0.643 (sensitivity 87.5%, specificity 39.3%, *P* = 0.078, **Figure 1A**). Patients were divided into a high PAR group (PAR > 7.40 × 10^9, n = 26, 50.0%) and a low PAR group (PAR ≤ 7.40 × 10^9, n = 26, 50.0%) according to the critical value of postoperative PAR. PAR was associated with lymph node metastasis (*P* = 0.008), pTNM stage (*P* = 0.011) and the presence of recurrence or metastasis (*P* = 0.005). PAR > 7.40 × 10^9 was positively correlated with patients having lymph node metastasis, advanced pTNM stage, and disease recurrence or metastasis.

### 3.3 Correlation of PAR and clinicopathologic characteristics

The high PAR group was 57.1% and 35.3% in male and female patients, respectively, indicating that PAR was more likely to be elevated in male patients but not statistically different (*P* = 0.139). The high PAR group was 80.0% and 46.8% of the positive and negative margins, respectively, indicating that PAR was more likely to be elevated in patients with positive margins, but with no statistically significant difference (*P* = 0.158). In the high PAR group, 60.0% of patients had a smoking history, and 40.7% of patients who had never smoked, indicating that PAR was more likely to be elevated in smoking patients, but the difference was not statistically significant (*P* = 0.165). When the tumor max diameter > 4cm and ≤ 4cm, the high PAR group was 61.1% and 44.1%, respectively, indicating that PAR was more likely to be elevated in larger tumors, but not significantly different (*P* = 0.244). The relationships between clinicopathological features and PAR are shown in **Table 2A**.

**Table 2A:** Relationship between clinical, pathological, and PAR in postoperative ASC patients.

Variables	N	Low PAR (%)	High PAR (%)	χ2	*P*-value
Gender				2.185	0.139
Male	35	15 (42.9)	20 (57.1)		
Female	17	11 (64.7)	6 (35.3)		
Age at diagnosis (years)				0.000	1.000
≤ 60	18	9 (50.0)	9 (50.0)		
> 60	34	17 (50.0)	17 (50.0)		
Smoking history				1.926	0.165
Yes	25	10 (40.0)	15 (60.0)		
No	27	16 (59.3)	11 (40.7)		
Differentiation				0.115	0.734
Poor	41	20 (48.8)	21 (51.2)		
Moderate-poor	11	6 (54.5)	5 (45.5)		
Tumor site				1.359	0.244
Upper	34	15 (44.1)	19 (55.9)		
Lower-middle	18	11 (61.1)	7 (38.9)		
Maximum tumor diameter (cm)				1.359	0.244
≤ 4	34	19 (55.9)	15 (44.1)		
> 4	18	7 (38.9)	11 (61.1)		
Margin				1.991	0.158
negative	47	25 (53.2)	22(46.8)		
positive	5	1 (20.0)	4 (80.0)		
T stage				0.078	0.780
T1a-T2a	29	14 (48.3)	15 (51.7)		
T2b-T4	23	12 (52.2)	11 (47.8)		
N stage				9.547	**0.008**
N0	36	23 (63.9)	13 (36.1)		
N1	13	3 (23.1)	10 (76.9)		
N2+N3	3	0 (0)	3 (100)		
pTNM Stage				6.470	**0.011**
IA1-IIA	31	20 (64.5)	11 (35.5)		
IIB-IIIB	21	6 (28.6)	15 (71.4)		
Survival				11.143	**0.001**
Yes	28	20 (71.4)	8 (28.6)		
No	24	6 (25.0)	18 (75.0)		
Recurrent or Metastatic				7.879	**0.005**
Yes	30	10 (33.3)	20 (66.7)		
No	22	16 (72.7)	6 (27.3)		

PAR, platelet to albumin ratio; ASC, lung adenosquamous carcinoma; pTNM: pathological tumor, node, metastasis staging.

Bold values provided is considered to be a statistically significant difference.

### 3.4 Optimal cut-off value for the HALP score

The X-tile software conducts statistical tests by using the enumeration method to group different values as the cut-off values, and the smallest *P*-value is considered to be the best cut-off value. The OS of 42 months as the cut-off was divided into two groups, and calculated by the X-tile software the optimal cut-off value for the HALP score before surgery was 24.3, at which point *P* = 0.0012 (**Figure 1B**). The optimal cut-off value for the postoperative HALP score was 19.8, at which point *P* = 0.1571 (**Figure 1C**). With DFS of 36 months as cut-off divided into two groups, the optimal preoperative HALP score was 24.3, when *P* < 0.0001 (**Figure 1D**). The optimal cut-off value for the HALP score after surgery was 19.8, at which point *P* = 0.1571 (**Figure 1E**).

### 3.5 Correlation of HALP score and clinicopathologic characteristics

Based on the best preoperative HALP score, the enrolled patients were divided into a high HALP group (HALP > 24.3, n = 46, 88.5%) and a low HALP group (HALP ≤ 24.3, n = 6, 11.5%). Preoperative HALP score was correlated with tumor recurrence or metastasis (*P* = 0.026) and age (*P* = 0.058), and HALP ≤ 24.3 was positively associated with disease recurrence or metastasis in patients. The low HALP group was 22.2% and 5.9% at tumor max > 4cm and ≤ 4cm, respectively, indicating that HALP score may be lower at larger tumors, but with no statistically significant difference (*P* = 0.079). At poorly and moderate-poorly differentiated tumors, the low HALP group was 14.6% and 0% respectively, indicating that HALP score was potentially low at poorly differentiated tumor, but there was no statistically significant difference (*P* = 0.177). The relationships between the clinicopathological features and HALP score are shown in **Table 2B**.

**Table 2B:** Relationship between clinical, pathological, and HALP score in postoperative ASC patients.

Variables	N	Low HALP score (%)	High HALP score (%)	χ2	*P*-value
Gender				0.001	0.972
Male	35	4 (11.4)	31 (88.6)		
Female	17	2 (11.8)	15 (88.2)		
Age at diagnosis (years)				3.591	**0.058**
≤ 60	18	0 (0)	18 (100)		
> 60	34	6 (17.6)	28 (82.4)		
Smoking history				0.591	0.442
Yes	25	2 (8.0)	23 (92.0)		
No	27	4 (14.8)	23 (85.2)		
Differentiation				1.820	0.177
Poor	41	6 (14.6)	35 (85.4)		
Moderate-poor	11	0 (0)	11 (100)		
Tumor site				0.709	0.400
Upper	34	3 (8.8)	31 (91.2)		
Lower-middle	18	3 (16.7)	15 (83.3)		
Maximum tumor diameter (cm)				3.079	0.079
≤ 4	34	2 (5.9)	32 (94.1)		
> 4	18	4 (22.2)	14 (77.8)		
Margin				0.388	0.533
negative	47	5 (10.6)	42(89.4)		
positive	5	1 (20.0)	4 (80.0)		
T stage				1.384	0.239
T1a-T2a	29	2 (6.9)	27 (93.1)		
T2b-T4	23	4 (17.4)	19 (82.6)		
N stage				1.591	0.451
N0	36	4 (11.1)	32 (88.9)		
N1	13	1 (7.7)	12 (92.3)		
N2+N3	3	1 (33.3)	2 (66.7)		
pTNM Stage				0.140	0.708
IA1-IIA	31	4 (12.9)	27 (87.1)		
IIB-IIIB	21	2 (9.5)	19 (90.5)		
Survival				7.913	**0.005**
Yes	28	0 (0)	28 (100)		
No	24	6 (25.0)	18 (75.0)		
Recurrent or Metastatic				4.974	**0.026**
Yes	30	6 (20.0)	24 (80.0)		
No	22	0 (0)	22 (100)		

HALP score, hemoglobin, albumin, lymphocyte, and platelet score; ASC, lung adenosquamous carcinoma; pTNM, pathological tumor, node, metastasis staging.

Bold values provided is considered to be a statistically significant difference.

### 3.6 Prognostic factors affecting OS in postoperative ASC patients

In univariate analysis, the cut margin (*P* = 0.013), the degree of differentiation (*P* = 0.021), N stage (*P* = 0.049), △Plt (*P* = 0.010), △Alb (*P* = 0.016), PAR (*P* = 0.003), NLR (*P* = 0.025), PLR (*P* = 0.029), preoperative HALP score (*P* = 0.000) and postoperative HALP score (*P* = 0.010) were all associated with the postoperative OS in ASC patients. Multivariate analysis showed that the cut margin (*P* = 0.461), N stage (*P* = 0.484), △Plt (*P* = 0.712), △Alb (*P* = 0.699), PLR (*P* = 0.329), NLR (*P* = 0.325) and postoperative HALP score (*P* = 0.673) were not significantly associated with the OS of postoperative ASC patients, but PAR (HR: 6.877, 95%CI: 1.817-26.038, *P* = 0.005), degree of differentiation (HR: 0.059, 95%CI: 0.006-0.591, *P* = 0.016), and preoperative HALP score (HR: 0.224, 95%CI: 0.068-0.733, *P* = 0.013) had significant effect on OS (**Table 3**).

**Table 3:** Univariable and multivariable cox regression analyses of OS.

OS: Cox regression analysis (N = 52, 24 death events)				
	univariable model		multivariable model	
Variables	HR (95% CI)	*P*-value	HR (95% CI)	*P*-value
Gender (male vs. female)	0.675	0.384		
	(0.279-1.635)			
Age (≤ 60 vs. > 60)	1.574	0.315		
	(0.650-3.812)			
Smoking history (yes vs. no)	0.859	0.712		
	(0.384-1.921)			
Differentiation (poor vs. moderate-poor)	0.091	**0.021**	0.059	**0.016**
	(0.012-0.694)		(0.006-0.591)	
Tumor site (upper vs. lower-middle)	0.806	0.632		
	(0.334-1.947)			
Maximum tumor diameter (≤ 4 vs. > 4)	1.943	0.106		
	(0.868-4.351)			
Margin (negative vs. positive)	3.705	**0.013**	1.619	0.461
	(1.325-10.363)		(0.450-5.827)	
T stage (T1a-2a vs. T2b-4)	1.331	0.487		
	(0.594-2.982)			
N stage (N0 vs. N1 vs. N2+N3)	1.993	**0.049**	0.743	0.484
	(1.004-3.957)		(0.324-1.705)	
pTNM Stage (IA1-IIA vs. IIB-IIIB)	1.129	0.767		
	(0.505-2.526)			
△Plt (≤ 62×10^9 vs. > 62×10^9)	2.995	**0.010**	1.206	0.712
	(1.300-6.902)		(0.448-3.248)	
△Alb (≤ -5.1 vs. > -5.1)	2.733	**0.016**	0.806	0.699
	(1.205-6.200)		(0.271-2.398)	
PAR (high vs. low)	4.064	**0.003**	6.877	**0.005**
	(1.606-10.284)		(1.817-26.038)	
PLR (high vs. low)	3.853	**0.029**	0.307	0.329
	(1.148-12.932)		(0.029-3.289)	
NLR (high vs. low)	4.029	**0.025**	3.047	0.325
	(1.191-13.632)		(0.332-27.995)	
Preoperative HALP score (high vs. low)	0.172	**0.000**	0.224	**0.013**
	(0.066-0.443)		(0.068-0.733)	
Postoperative HALP score (high vs. low)	0.295	**0.010**	0.737	0.673
	(0.117-0.745)		(0.179-3.034)	

OS, overall survival; pTNM, pathological tumor, node, metastasis staging; △Plt, the platelet difference before and after surgery; △Alb, the albumin difference before and after surgery; PAR, platelet to albumin ratio; PLR, platelet to lymphocyte ratio; NLR, neutrophil to lymphocyte ratio; preoperative HALP score, preoperative hemoglobin, albumin, lymphocyte, and platelet score; postoperative HALP score, postoperative hemoglobin, albumin, lymphocyte, and platelet score.

Bold values provided is considered to be a statistically significant difference.

### 3.7 Prognostic factors affecting DFS in postoperative ASC patients

Univariate Cox analysis showed that the resection margin (*P* = 0.029), the degree of differentiation (*P* = 0.045), maximum tumor diameter (*P* = 0.018), N stage (*P* = 0.035), △Plt (*P* = 0.007), △Alb (*P* = 0.007), PAR (*P* = 0.004), NLR (*P* = 0.041), PLR (*P* = 0.030), preoperative HALP score (*P* = 0.000) and postoperative HALP score (*P* = 0.011) were all associated with postoperative DFS in ASC patients. Multivariate analysis showed that the maximum tumor diameter (HR: 3.442, 95%CI: 1.148-10.318, *P* = 0.027) and preoperative HALP score (HR: 0.268, 95%CI: 0.085-0.847, *P* = 0.025) had significant influence on DFS (**Table 4**).

**Table 4:** Univariable and multivariable cox regression analyses of DFS.

DFS: Cox regression analysis (N = 52, 30 recurrent or metastatic events)				
	univariable model		multivariable model	
Variables	HR (95% CI)	*P*-value	HR (95% CI)	*P*-value
Gender (male vs. female)	0.780	0.583		
	(0.322-1.891)			
Age (≤ 60 vs. > 60)	1.440	0.419		
	(0.595-3.481)			
Smoking history (yes vs. no)	1.078	0.854		
	(0.483-2.408)			
Differentiation (poor vs. moderate-poor)	0.128	**0.045**	0.129	0.079
	(0.017-0.952)		(0.013-1.272)	
Tumor site (upper vs. lower-middle)	0.911	0.836		
	(0.377-2.204)			
Maximum tumor diameter (≤ 4 vs. > 4)	2.665	**0.018**	3.442	**0.027**
	(1.180-6.019)		(1.148-10.318)	
Margin (negative vs. positive)	3.126	**0.029**	1.247	0.746
	(1.126-8.678)		(0.328-4.742)	
T stage (T1a-2a vs. T2b-4)	1.639	0.228		
	(0.734-3.660)			
N stage (N0 vs. N1 vs. N2+N3)	2.016	**0.035**	0.739	0.443
	(1.051-3.866)		(0.341-1.600)	
pTNM Stage (IA1-IIA vs. IIB-IIIB)	1.201	0.657		
	(0.535-2.693)			
△Plt (≤ 62×10^9 vs. > 62×10^9)	3.129	**0.007**	2.332	0.109
	(1.360-7.202)		(0.827-6.574)	
△Alb (≤ -5.1 vs. > -5.1)	3.106	**0.007**	1.573	0.446
	(1.370-7.042)		(0.490-5.047)	
PAR (high vs. low)	3.922	**0.004**	2.772	0.114
	(1.549-9.933)		(0.783-9.812)	
PLR (high vs. low)	3.819	**0.030**	0.571	0.665
	(1.136-12.835)		(0.045-7.232)	
NLR (high vs. low)	3.575	**0.041**	1.469	0.750
	(1.057-12.096)		(0.138-15.655)	
Preoperative HALP score (high vs. low)	0.126	**0.000**	0.268	**0.025**
	(0.046-0.347)		(0.085-0.847)	
Postoperative HALP score (high vs. low)	0.299	**0.011**	1.113	0.887
	(0.118-0.756)		(0.254-4.886)	

DFS, disease-free survival; pTNM, pathological tumor, node, metastasis staging; △Plt, the platelet difference before and after surgery; △Alb, the albumin difference before and after surgery; PAR, platelet to albumin ratio; PLR, platelet to lymphocyte ratio; NLR, neutrophil to lymphocyte ratio; preoperative HALP score, preoperative hemoglobin, albumin, lymphocyte, and platelet score; postoperative HALP score, postoperative hemoglobin, albumin, lymphocyte, and platelet score.

Bold values provided is considered to be a statistically significant difference.

### 3.8 Relationship between OS and PAR, preoperative HALP score and differentiation degree in postoperative ASC patients

**Figure 2:**
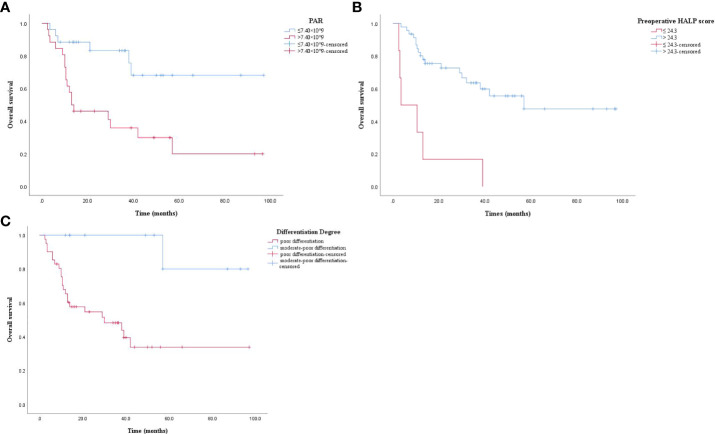
**(A)** Kaplan-Meier survival curve. PAR > 7.40 × 10^9 had a lower OS (*P* = 0.001). OS: overall survival; PAR: platelet to albumin ratio. **(B)** Kaplan-Meier survival curve. Preoperative HALP score ≤ 24.3 had a lower OS (*P* = 0.000). OS: overall survival; preoperative HALP score: preoperative hemoglobin, albumin, lymphocyte, and platelet score. **(C)** Kaplan-Meier survival curve. Poor differentiation had a lower OS (*P* = 0.004). OS: overall survival.

The results of Kaplan-Meier analysis were verified by log-rank test that patients with high PAR level had significantly lower OS than those with low PAR levels (*P* = 0.001, **Figure 2A**). The OS of patients with low HALP score before surgery was significantly lower than those with high HALP score (*P* = 0.000, **Figure 2B**). The OS of poorly differentiated ASC patients was significantly lower than that in moderately poorly differentiated patients (*P* = 0.004, **Figure 2C**).

### 3.9 Association between DFS and PAR, preoperative HALP score, tumor maximum diameter and degree of differentiation in postoperative ASC patients

**Figure 3:**
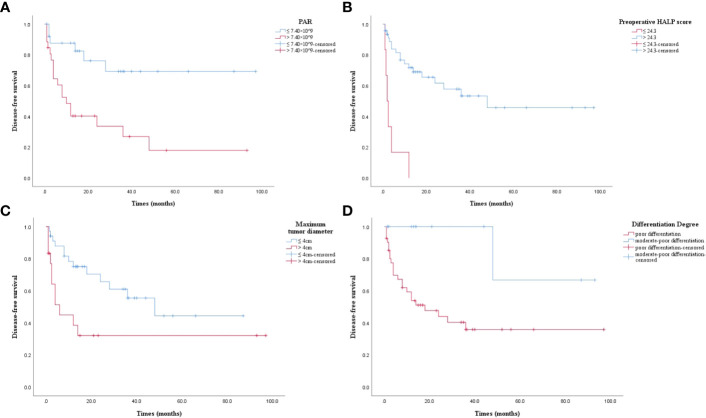
**(A)** Kaplan-Meier survival curve. PAR > 7.40 × 10^9 had a lower DFS (*P* = 0.002). DFS: disease-free survival; PAR: platelet to albumin ratio. **(B)** Kaplan-Meier survival curve. Preoperative HALP score ≤ 24.3 had a lower DFS (*P* = 0.000). DFS: disease-free survival; preoperative HALP score: preoperative hemoglobin, albumin, lymphocyte, and platelet score. **(C)** Kaplan-Meier survival curve. Maximum tumor diameter > 4cm had a lower DFS (*P* = 0.013). DFS: disease-free survival. **(D)** Kaplan-Meier survival curve. Poor differentiation had a lower DFS (*P* = 0.016). DFS: disease-free survival.

The results of Kaplan-Meier analysis were confirmed by the log-rank test. DFS was significantly lower in high PAR patients than in those with low PAR level (*P* = 0.002, **Figure 3A**). DFS was significantly lower in patients with low HALP score than patients with high HALP score (*P* = 0.000, **Figure 3B**). DFS in patients with maximum tumor diameter > 4cm was significantly lower than those with maximum tumor diameter ≤ 4cm (*P* = 0.013, **Figure 3C**). Poorly differentiated ASC patients had significantly lower DFS than those with moderate-poor differentiation (*P* = 0.016, **Figure 3D**).

## 4 Discussion

This study suggests that the PAR, degree of tumor differentiation, and preoperative HALP score are independent prognostic factors for postoperative OS in ASC patients, and that the maximum tumor diameter and preoperative HALP score are independent prognostic factors for postoperative DFS in patients with ASC. Liu et al. retrospectively studied the prognostic value of 190 patients with stage I NSCLC who underwent radical surgery. The results showed that NLR (*P* = 0.016), RDW-SD (*P* = 0.004), CEA (*P* = 0.011), and lymphovascular invasion (*P* = 0.027) were independent risk factors for postoperative outcomes in stage I NSCLC patients, and combination therapy had predictive value (18). However, in our study, univariate analysis showed that NLR was associated with postoperative OS (*P* = 0.025) and DFS (*P* = 0.041) in ASC patients, but multivariate analysis showed that NLR was not an independent prognostic factor in ASC patients, which may be related to small sample size, further tumor classification and lack of genetic testing. Therefore, a large number of prospective studies are still needed to verify whether inflammation and inflammation-derived indicators have an impact on the prognosis of patients with ASC.

Many studies have demonstrated that inflammation plays an important role in the initiation, progression and metastasis of solid tumors, which in turn can induce inflammation, thus forming a “snowball” effect in a vicious cycle (19). Platelet is an important factor in the coagulation system. In addition to hemostasis and coagulation, platelets are also involved in inflammation and tumor progression. Platelets can protect tumor cells from these injuries by covering them (20). Platelets can also stimulate the proliferation of tumor cells and adhere to other cells by the secretion of vascular endothelial growth factor (VEGF) (21). Albumin is an objective indicator reflecting the nutritional status of tumor patients (22), which can also reflect the degree of inflammation in the body to a certain extent. Low albumin level is closely related to the size and aggressiveness of tumors (23). At the same time, increasing evidence shows that albumin can be used in the early diagnosis, prognosis or prediction of solid malignant tumors (24). Hemoglobin is an important indicator to determine whether anemia is present or not. Studies found that the relative risk of death in patients with anemia in lung cancer, head and neck cancer, prostate cancer, and lymphoma increased by 19% (95%CI, 10-29%), 75% (37-123%), 47% (21-78%), and 67% (30-113%), respectively. The overall estimated risk was increased by 65% (54-77%), indicating that anemia was associated with reduced survival in some malignancies (25). At the same time, anemia stimulates the kidney to produce erythropoietin (EPO), which can stimulate erythropoiesis, thus improving the anemia status. However, increasing evidences indicate that the coexpression of EPO and EPO receptors is thought to be associated with tumor cell growth, invasion, and metastasis (26–28). Aboouf et al. (29) found that the erythropoietin receptor regulated NO production by controlling iNOS expression and AKT phosphorylation, and, in turn, pAKT and iNOS used NO to regulate mitochondrial biogenesis in cancer and stromal cells. In a xenograft model mimicking EPO treatment in lung cancer patients, knockdown of EPOR impaired tumor growth, cellular respiration, mitochondrial content, and iNOS expression and AKT phosphorylation of lung cancer xenografts. Studies showed that functional EPO receptor signaling was critical for the tumor-promoting growth effects of EPO through *in vitro* and *in vivo* experiments, indicating that EPOR expression was positively correlated with the expression of the mitochondrial marker VDAC1 in human NSCLC patient biopsies. The tumor microenvironment (TME) is a complex environment, in which tumor cells live and develop, consisting of immune cells and stromal cells, playing a crucial role in tumor progression, metastasis, and response to therapy (30). The interaction between tumor cells and infiltrating immune cells is complex and multifaceted, and the role of tumor cells on the metabolic activation of infiltrating immune cells can enable tumor cells to adapt to the metabolic activities of infiltrating immune cells during growth (31). The metabolic characteristics of the microenvironment can also affect the development of tumor immunosuppression, which is mainly related to the differentiation process of T cells to regulate T cells, which can promote T cells inactivation, restrain production of anti-inflammatory cytokines and expression of immune checkpoint molecules (32). The HALP score combines hemoglobin, albumin, lymphocyte and platelet counts, reflecting the degree of anemia, nutritional status, immune status and coagulation function, which is a comprehensive score. This score has been reported to be a good prognostic marker for a variety of cancers (16, 17, 33, 34). Vlatka et al. (33) retrospectively analyzed the prognostic value of 153 newly diagnosed patients with diffuse large B cell lymphoma, and the results showed that five-year OS (47.3% *vs* 79.5%, *P <* 0.001) and five-year EFS (40.6% *vs* 76.7%, *P <* 0.001) were significantly worse when the patient’s HALP score ≤ 20.8. The study of Güç et al. (34) showed that the HALP score could predict the prognosis of patients with advanced NSCLC, and the critical point of HALP score was 23.24 (AUC = 0.928; 95%CI: 0.901-0.955, *P* < 0.001). Multivariate analysis showed that low HALP score (HR = 2.988, 95%CI: 2.065-4.324, P < 0.001) was an independent factor associated with reduced incidence of OS. As one of the derived indicators of peripheral blood inflammatory, PAR has clinical value in predicting the prognosis of some cancer patients (10, 35–40). In addition, the platelet count and serum albumin are easily detected and of low cost.

Several recent studies have reported the prognostic effect of PAR in patients with malignant tumors, including NSCLC (10), esophageal squamous cell carcinoma (35), bile duct carcinoma (36), pancreatic ductal adenocarcinoma (37), urothelial carcinoma (38), colorectal cancer (39), and hepatocellular carcinoma (40). In addition, a recent study by Tan et al. suggested that PAR may serve as a new prognostic predictor of IgA nephropathy (41). Another study showed that PAR was a novel and reliable indicator of disease activity in axial arthritis of the spine and had high diagnostic value for active axial arthritis of the spine (42).

All of these studies are consistent with our findings. In addition, the optimal cut-off value for prognosis of PAR and HALP score varies in different reports, and the underlying mechanism is unknown. Tumor differences in biological behavior, different sample size, cohort characteristics, ethnic differences, and population heterogeneity may be potential explanations for the inconsistent results. Therefore, more large-scale studies are urgently needed to validate this conclusion.

The limitations of this study are as follows: First, it was a retrospective study with bias, a small sample size and a large time span. Secondly, this study did not include tumor-related indicators, albumin to globulin ratio, and lymphocyte to monocyte ratio, which will be further analyzed in subsequent studies. Finally, in our study, no validation cohort was included to verify our results. In conclusion, this study suggests that PAR, preoperative HALP score, and the degree of tumor differentiation are independent prognostic factors for OS in patients with ASC after radical surgery, and that maximum tumor diameter and preoperative HALP score are independent prognostic factors for postoperative DFS in ASC patients, but it still needs to be confirmed by a large number of randomized multicenter, large sample, prospective trials.

## Author contributions

TZ and WL collected and extracted data to judge the eligibility of the study. TZ analyzed the data and wrote the article. CX designed the study and revised the article. All authors contributed to the article and approved the submitted version.
